# A Quality Improvement Project (QIP) Within the Bolton Learning Disability (LD) Team (Greater Manchester Mental Health Trust) Aiming to Enhance Communication Among Multi-Disciplinary Team (MDT) Professionals and Wider Stakeholders, for the Purposes of Enhanced Care Delivery and Improved Patient Outcomes During COVID-19 pandemic. Project Lead: Dr Syeda Hasan

**DOI:** 10.1192/bjo.2022.301

**Published:** 2022-06-20

**Authors:** Syeda Hasan, Rachel Moir, Jessica Neary, Rupa Gupta

**Affiliations:** 1 Pennine Care NHS Foundation Trust, Manchester, United Kingdom; 2 Greater Manchester Mental Health Trust, Manchester, United Kingdom

## Abstract

**Aims:**

This QIP aims to improve communication and information sharing between the community LD team, administration team, service providers and wider stakeholders, to ensure patient safety. The primary objective is to evaluate local initiatives to improve communication between MDT professionals and wider stakeholders. The secondary objective is to improve patient safety and staff satisfaction.

**Methods:**

The COVID-19 pandemic created unprecedented communication challenges within the workforce and highlighted areas requiring review; this included information sharing among internal and external teams, collaborative teamworking, support in absence of senior clinical leadership and transition pathways from Child and Adolescent Mental Health Services to adult LD services. The QIP was initiated in March 2021.

The discovery process included an initial consultation exploring practitioners’ experiences, areas for development and to share ideas for good practice.

We used QI methodology, following ‘plan-do-study-action’ cycles, to analyse change. Change ideas included a single point of contact for internal and external queries, regular complex case management meetings, development of a referral process and clinical review for complex cases along with teaching sessions.

Qualitative feedback from the team pre- and post-intervention, at baseline and regular follow-up intervals, in the form of monthly team meetings, emails, focus-groups and semi-structured interviews.

**Results:**

A full thematic map was created after initial consultation; themes included communication improvement between teams and external agencies, timely support for complex case management, improving transition processes and development of robust clinical review processes.

Qualitative feedback has been collated, analysed and final recommendations to be shared with the MDT professionals

**Conclusion:**

Preliminary results have shown improvements in communication among the MDT, stakeholders, and external agencies.

The consultation process highlighted that there is a substantial need for standardisation and consistency within communicative practices to promote enhanced care delivery and improved patient outcomes.

